# Complete Genome Sequence of the Telford Type S Strain of Mycobacterium avium subsp. paratuberculosis

**DOI:** 10.1128/MRA.00004-19

**Published:** 2019-03-14

**Authors:** Rudiger Brauning, Karren Plain, Milan Gautam, Tonia Russell, C. Carolina Correa, Patrick Biggs, Richard Whittington, Alan Murray, Marian Price-Carter

**Affiliations:** a AgResearch Ltd., Invermay Agricultural Centre, Mosgiel, New Zealand; b University of Sydney, Camden, New South Wales, Australia; c School of Veterinary Science, EpiCentre, Massey University, Palmerston North, New Zealand; d Ramaciotti Centre for Genomics, University of New South Wales (UNSW), Sydney, Australia; e Infectious Disease Research Centre, School of Veterinary Science, Hopkirk Research Institute, and School of Fundamental Sciences, Massey University, Palmerston North, New Zealand; f School of Veterinary Science, Hopkirk Research Institute, Massey University, Palmerston North, New Zealand; g AgResearch Ltd., Hopkirk Research Institute, Palmerston North, New Zealand; Broad Institute of MIT and Harvard University

## Abstract

Mycobacterium avium subsp. paratuberculosis is the causative agent of Johne’s disease (JD). Here, we report the complete genome sequence of Telford 9.2, a well-characterized representative strain of the M. avium subsp. paratuberculosis S subtype that is endemic in New Zealand and Australian sheep.

## Abstract (precis)

Mycobacterium avium subsp. paratuberculosis is the causative agent of Johne’s disease (JD).

## ANNOUNCEMENT

Mycobacterium avium subsp. paratuberculosis is the causative agent of Johne’s disease (JD), a chronic, generally subclinical but sometimes fatal granulomatous enteritis of ruminants (1). M. avium subsp. paratuberculosis subtype S (also called either subtype I or subtype III) has been isolated primarily from sheep but also from other ruminant species (reviewed in reference 2). Only draft genomes (3–5) of M. avium subsp. paratuberculosis type S are currently available. Here, we announce the complete genome sequence of Telford 9.2, an IS*1311* type S IS*900* restriction fragment length polymorphism (RFLP) type S1 strain. This is a clonal culture (passage level 5, including its primary isolation from sheep feces) of an isolate from a clinically infected sheep from New South Wales, Australia. It has been used as inoculum in an experimental model for clinical JD in sheep (6, 7), characterized genetically (8), and is representative of the M. avium subsp. paratuberculosis type endemic in Australian and New Zealand (NZ) sheep (9–11).

For Illumina and PacBio sequencing, bacterial stock was inoculated into either supplemented Middlebrook 7H9 (12) (Illumina) or M7H9C (PacBio) (13) liquid medium, cultured for 3 to 4 weeks, and then cultivated on modified Middlebrook 7H10 solid medium (12), harvested, and stored at −80°C.

Genomic DNA was prepared for both PacBio and Illumina sequencing by isopropanol precipitation and 70% ethanol wash of cetyltrimethylammonium bromide (CTAB)/phenol-chloroform-extracted cellular material after stepwise enzymatic digestion with lysozyme, RNase A, and proteinase K. For PacBio sequencing, the DNA was also digested with mutanolysin prior to proteinase K digestion and subjected to extra cleanup and concentration on Ampure PB magnetic beads.

A PacBio library was constructed and sequenced at the Ramaciotti Centre in Sydney, Australia, using P6-C4 chemistry, and sequenced to a coverage depth of 80× on the PacBio RS II platform on a single-molecule real-time (SMRT) cell. It was improved with Illumina MiSeq 250-bp paired-end (PE) reads generated by sequencing two cultures of the Telford 9.2 reference strain. MiSeq-indexed libraries were created at New Zealand Genomics Limited using Nextera XT DNA kits (Illumina, San Diego, CA). Average coverage was 120× from PacBio data and 135× from Illumina data. There were 2.5 million Illumina PE reads (909 Mbp) and 150,000 PacBio reads prefilter (555 Mbp; *N*_50_ value, 10.5 kbp). PacBio reads went through default filtering steps in SMRTPipe v1.87.139483, which reduced read numbers to 63,000 (491 Mbp; *N*_50_ value, 10.8 kbp), and were assembled using PacBio Hierarchical Genome Assembly Process v3 (HGAP3; SMRT analysis v2.3.0) into a single contig (Telford1) of the size expected for a complete M. avium subsp. paratuberculosis genome and with a GC content of 69.2%, which is typical of M. avium subsp. paratuberculosis (3–5, 14). The PacBio-based assembly was improved by removing a 9-kbp overlap between the start and the end of the genome, orienting the genome with the start position at the beginning of the *dnaA* gene and mapping Illumina reads onto the PacBio assembly to detect and repair small-scale variations, as described in Table 1.

**TABLE 1:** Discrepancies between Illumina and PacBio data^a^

Position before fix	Variant type	Accepted solution	PacBio allele	Illumina allele	Applied fix
780880^b^	Indel	Illumina	T	TG	Insertion
931746	SNP	PacBio	C	G	na
1112469	Indel	Illumina	GCCCCC	GCCCCCC	Insertion
1302183	Indel	Illumina	AGGGG	AGGGGG	Insertion
1969375	Indel	Illumina	GCCCCC	GCCCCCC	Insertion
2128150	Indel	Illumina	ACCCCC	ACCCCCC	Insertion
2276090	Indel	Illumina	CGGGGG	CGGGGGG	Insertion
2577759	SNP	PacBio	G	A	na
2635929	Indel	Illumina	GCCCC	GCCCCC	Insertion
2642118	SNP	PacBio	C	T	na
2705636	Indel	Illumina	GCCCCC	GCCCCCC	Insertion
3024648	Indel	Illumina	T	TC	Insertion
3201490	SNP	PacBio	C	G	na
3201602	SNP	PacBio	A	G	na
3211597	Indel	Illumina	CGGGGGGG	CGGGGGGGG	Insertion
3450836	Indel	PacBio	CATCGTCGCGCCGTGCTGGGCGGCCAGCGCGTCGCCGACCAGGCTGCGCGCCGGCTCGACGCGCCGCGCGGCCCGCAGCGCCTGCTGGG	C	na
4313098	SNP	Illumina	N	G	Base change
4314018	Indel	Illumina	GTTT	GTT	Deletion
4318473	Indel	Illumina	AC	A	Deletion
4319018	Indel	Illumina	AC	A	Deletion
4319236	Indel	Illumina	GTTT	GTT	Deletion
4319286	Indel	Illumina	CGGGG	CGGG	Deletion
4320148	Indel	Illumina	ACGCGCGC	ACGCGC	Deletion
4371898	SNP	PacBio	G	T	na
4416918	Indel	PacBio	CCGTTCGGCGCCGAGCGTCACGCCAGCGTGGCGCTCGCGGGCCGGCGCCACGCTGGCGTGACG	CCG	na
4421523	Indel	Illumina	GCCCC	GCCCCC	Insertion
4572001	SNP	PacBio	G	A	na
4594338	Indel	Illumina	ACCCC	ACCCCC	Insertion

a Illumina reads were mapped onto the PacBio assembly using BWA-MEM (17) v0.7.17-r1188 with parameter “-M,” and then variants (SNPs and indels) were detected (SAMtools [18] v1.3 with parameters “view -q 30 -F 256,” SAMtools v1.3 with parameters “mpileup -t DP,AD,” BCFtools v0.1.16 with parameters “call –cv,” BCFtools v0.1.16 with parameters “view -M2”). For each variant, a read depth greater than 10 was required, and a visual check of mapq values as well as the reference and alternative allele counts was performed. As a result of this analysis, for SNPs the PacBio alleles were accepted, for short indels the Illumina alleles were accepted, and for longer indels the PacBio alleles were accepted. All variants were verified by comparing 200 bp of flanking sequence (centered on the variants) to very closely related map strains (3, 4) using the “map to a reference” function in Geneious (19) and also comparing this fragment to M. avium subsp. paratuberculosis strains included in NCBI taxid 1770 using the NCBI BLAST service with default settings. SNP, single nucleotide polymorphism; na, no action.

b For the indel at position 780880, the Telford1 sequence differed from closely related strains in both PacBio and Illumina alleles; Sanger sequencing confirmed the Illumina call.

Telford1 has a sequence length of 4,907,428 bases, 4,377 coding sequences as predicted with the NCBI Prokaryotic Genome Annotation Pipeline (15), and an *in silico* IS*1311* type S IS*900* RFLP type S1 type (16).

### Data availability.

The genome assembly is available at GenBank under accession number CP033688 and the BioProject accession number PRJNA504678; raw data are available under SRA accession numbers SRX4997502 (Illumina) and SRX4997501 (PacBio), and *in silico* typing results can be found at https://doi.org/10.6084/m9.figshare.7635977.
